# Enhanced Durability of Wood Cutting Tools through Thermal Cycling

**DOI:** 10.3390/ma17205051

**Published:** 2024-10-16

**Authors:** Hamza Sofiane Meddas, Muftah Zorgani, Majid Heidari, Mousa Javidani, Tom Levasseur, Mohammad Jahazi

**Affiliations:** 1 Department of Mechanical Engineering, École de Technologie Superieure (ETS), Montreal, QC H3C 1K3, Canada; 2 DK SPEC Company, 1060, Chemin Olivier, St.-Nicolas, Levis, QC G7A 2M8, Canada; 3 Department of Applied Science, University of Quebec at Chicoutimi, Saguenay, QC G7H 2B1, Canada

**Keywords:** cold-work tool steel, double quenching, PAGS refinement, edge stability, PVD coating

## Abstract

This study investigates the impact of multi-step austenitization heat treatment on the in-service life of modified AISI A8 cold work tool steel knives used in wood cutting. The knives were subjected to two treatment methods: single quenching and double tempering (SQDT) and double quenching and double tempering (DQDT). Both treatments were followed by physical vapor deposition (PVD) coating to enhance surface properties. The DQDT treatment resulted in a finer microstructure and more uniform carbide distribution. Field tests on 24 knives over 124 h demonstrated up to 130% improvement in wear resistance for DQDT knives, along with superior edge stability and better PVD coating preservation. DQDT knives exhibited ductile fractures characterized by dimples, contrasting with the brittle fracture and cleavage facets in SQDT knives. Residual stress measurements showed higher compressive stresses in DQDT knives (−280 MPa) compared to SQDT knives (−30 MPa), which increased further after field testing. The enhanced performance of DQDT knives is attributed to their refined microstructure, improved carbide distribution, and higher compressive residual stresses, offering significant potential for improving wood cutting tool efficiency and durability.

## 1. Introduction

Wood processing industries rely heavily on high-performance cutting tools to maintain efficiency and productivity in their operations. These tools, particularly those used in chipping and canting, are subjected to extreme conditions including cyclic loading, high-speed impacts, and abrasive environments [1,2]. The demanding nature of these applications leads to significant wear and potential failure of the cutting edges, necessitating frequent tool replacements and resulting in increased operational costs and downtime. Consequently, there is a pressing need in the industry for cutting tools with enhanced edge stability, wear resistance, and overall durability.

The performance of wood cutting tools is intrinsically linked to the microstructural characteristics of the tool steels used in their fabrication. Research [3,4] has demonstrated that fine-grained structures with uniformly distributed spherical carbides exhibit superior wear resistance and toughness. The presence of retained austenite, when properly controlled, can also contribute to the surface wear and to the tool’s ability to withstand impact loads [5]. Moreover, the development of complex carbide structures, particularly during tempering in cold-work tool steels, has shown significant improvements in wear resistance [6,7]. Achieving an optimal balance between hardness and toughness is crucial, as excessive hardness can lead to brittleness and premature failure, while insufficient hardness results in rapid wear. Advanced techniques such as cryogenic treatment and surface engineering, including the application of hard coatings, have been explored to further enhance the microstructural properties and surface characteristics of tool steels [8,9].

AISI A8 is an air hardened cold-work tool steel which has gained significant attention in the wood processing industry due to its favorable combination of wear resistance and toughness [10]. This medium-carbon, high-chromium steel is characterized by its ability to maintain hardness at elevated temperatures, making it particularly suitable for high-speed cutting applications. The microstructure of AISI A8 typically consists of a tempered martensite matrix with dispersed secondary M_23_C_6_ carbides [11]. The presence of these carbides contributes to the wear resistance, and the tempered martensitic matrix provides the necessary toughness. While these microstructural features contribute to the mechanical resistance and the overall performance, a recent study conducted by Mbakop et al. [12] on AISI A8 tool steel indicated that this steel is it still susceptible to severe tool failure and suffers from edge stability in demanding wood cutting operations.

Thermomechanical processing routes have been conventionally used as a promising approach to enhance the mechanical properties and microstructural characteristics of tool steels. Pillai et al. [13] studied the effects of varying austenitization temperatures and quenching mediums on the tribological characteristics of AISI A8 steel. They reported that a higher dissolution of alloying elements in the matrix improves its wear resistance. However, a higher dissolution of alloying elements requires relatively high austenitization temperatures, and this could lead to abnormal grain growth [14]. For this purpose, non-conventional heat treatments, which often consist of multiple heating and cooling cycles, have emerged lately and have shown great efficiency in refining the microstructure while dissolving a specific fraction of alloying elements in the matrix [15]. It has been reported that the application of multiple heating and quenching cycles modified the carbide morphology [16,17], size [18], and distribution [19], as well as refining the overall grain size [20,21], resulting in concomitant enhancement of wear resistance [22,23] and impact toughness [24,25,26].

While the effects of coatings [27,28,29] and knife geometry [30,31] on the edge stability of cutting tools have been documented in the literature, very few data are available on the effect of the steel substrate on tool performance, especially in wood cutting applications. The present work has been defined in this context and aims to study the effect of thermal cycling on the wear resistance and toughness of an AISI A8 tool substrate used in the wood cutting industry.

The objective of this study was to determine a specific heat treatment cycle that would allow an optimized balance between hardness and toughness. Additionally, assessing the edge performance of wood cutting tools presents significant challenges due to the complex and dynamic nature of the wood cutting process. Traditional laboratory tests, despite their interest, often fail to fully replicate the harsh conditions encountered in industrial wood processing environments. Therefore, in the present work, field trials at the industrial scale were conducted to quantitatively assess the impact of microstructure modification on the performance of the cutting tools. The mechanisms governing microstructure evolution and their impact on wear and toughness are discussed.

## 2. Materials and Methods

The chemical composition of the AISI A8 alloy (ASTM A681 [32]) used in the present investigation is presented in Table 1. These compositional data were obtained using a SPECTROMAXx (LMX10, Kleve, Germany) arc/spark optical emission spectrometer. The alloy is a modified AISI A8 tool steel, as it has a higher content of Cr (8 wt.% instead of 5 wt.%) compared to the standard/conventional AISI A8. As a result, it is often called modified AISI A8, and this denomination will be used throughout the present work. The steel was supplied in the annealed condition and consisted of a ferritic matrix and primary M_23_C_6_ carbides.

**Figure 1:**
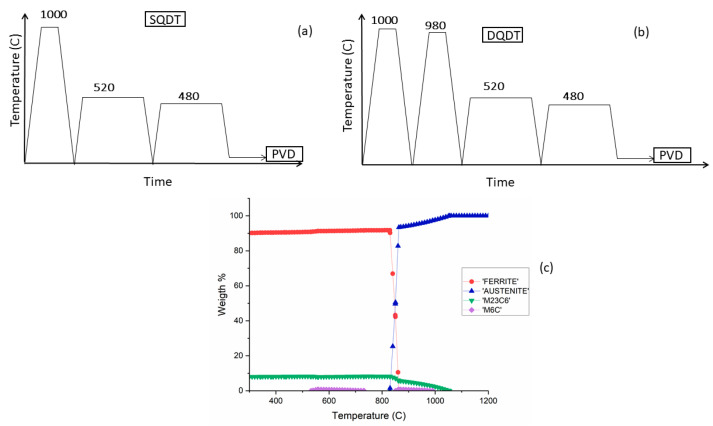
(**a**) Thermal cycle of SQDT; (**b**) thermal cycle of DQDT; and (**c**) phase diagram thermodynamic simulation of the modified A8 steel.

Two distinct heat treatments (HTs) were used: (1) single quenching double tempering (SQDT) treatment, consisting of one austenitization–quenching cycle followed by a double tempering cycle; and (2) the newly designed double quenching double tempering (DQDT) treatment, consisting of two austenitization–quenching cycles, followed by a double tempering cycle. The SQDT and DQDT HT cycles are presented in Figure 1a and Figure 1b, respectively. The HT parameters were selected based on a thermodynamic simulation calculated using the JMatPro^®^ v11 software [33], as shown in Figure 1c. The temperature for the first step of austenitization in the DQDT process must be chosen carefully to ensure incomplete carbide dissolution. Undissolved carbides play an important role during the second austenitization step, serving as nucleation sites for new austenite grains and pinning them to prevent grain growth, as reported in the literature [34]. As for the tempering cycle, a widespread practice in the thermal processing of tool steels often includes multiple tempering cycles. The first tempering cycle produces tempered martensite and allows secondary alloy carbide precipitation. During cooling from the first tempering, an indirect decomposition of retained austenite to fresh martensite could occur, and subsequent tempering cycles are used to temper the newly formed fresh martensite [10].

HTs were carried out using a vacuum industrial-size furnace (BMI horizontal gas quenching vacuum B8T furnace), with a thermocouple attached to a piece adjacent to the knives for temperature control. Following the HT, the physical vapor deposition (PVD) method was used to apply a multilayer coating on the surface of the knives.

For each HT condition (SQDT and DQDT), two test pieces with the same knife thickness were heat-treated alongside the knives to facilitate comprehensive microstructural characterization. These test pieces underwent conventional metallographic preparation techniques, including grinding up to P4000 followed by a final polishing step using a 1 µm diameter diamond suspension powder, and etching with 3% Nital to reveal the substrate microstructure. The prepared samples were then examined using an Olympus LEXT4100 laser (Shinjuku City, Japan) confocal microscope and a Hitachi TM3000 (Chiyoda City, Japan) scanning electron microscope (SEM) coupled with an energy dispersive spectrometer (EDS) to analyze grain structure, carbide distribution, and morphology. At least 10 micrographs for each condition were used for the measurement of prior austenite grain size (PAGS) and carbide size.

**Figure 2:**
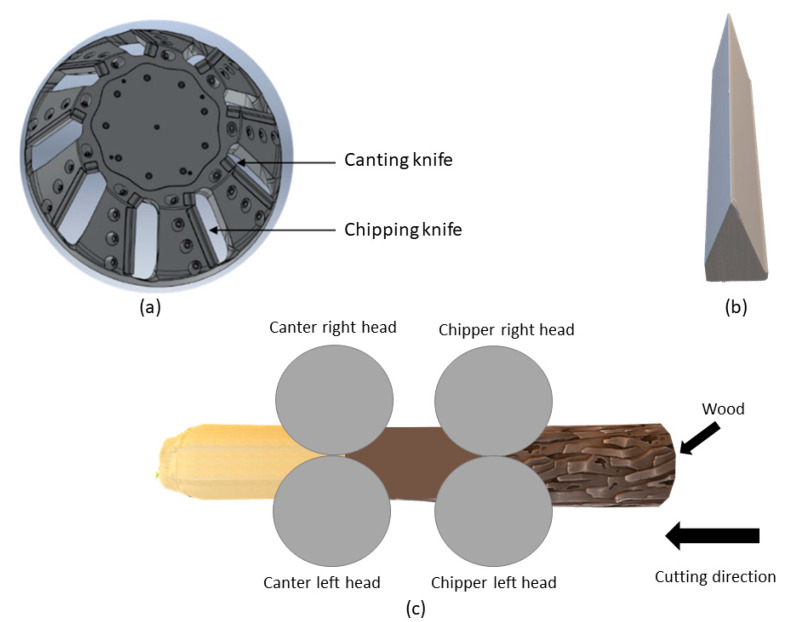
(**a**) Illustration of the chipper–canter head with respective knife placements; (**b**) side view of knife geometry; (**c**) schematic of wood cutting process showing the four chipper–canter heads used in the field tests.

The industrial field tests were designed to rigorously evaluate the performance of knives treated with SQDT and DQDT HTs. A total of 24 knives were tested, equally divided between the two treatments. These were strategically installed in four sets across four chipper–canter heads, composed of two canter heads (left and right) and two chipper heads (left and right), ensuring a comprehensive assessment of performance in different operational contexts. These machines are used in primary wood industries for processing small-diameter logs and they perform dual functions: the chipping knives sever slices of wood from the log, converting them into chips, while the canting knives smooth the surface of the resulting cant [1]. All knives operated continuously for 124 h, simulating extended industrial use. The chipper–canter head, the 3D-side view of the knife, and the industrial field test setting are illustrated in Figure 2a, Figure 2b, and Figure 2c, respectively.

The heat-treated and PVD-coated knives were analyzed using a novel approach recently developed by Mbakop et al. [12] to quantify the wear damage. This method involves high-precision 3D scanning of the knives before and after the field test using a digital microscope (Keyence VR-5200, Birmingham, AL, USA). The scans were used to create detailed 3D models of the knife edges, allowing for accurate measurement of volume loss due to wear. This non-destructive technique provides an assessment of the wear patterns and material removal across the entire cutting edge. Furthermore, the fractured surfaces of the damaged knives were observed under the LEXT microscope and underwent SEM for the analysis.

The u-X360 Pulstec machine, which employs the X-ray diffraction (XRD) technique for residual stress measurement, was also used to quantify the surface residual stresses. Measurements were performed on the knives before and after the field test.

This non-destructive method is based on measuring the strain in the crystal lattice and calculating the associated stress using the elastic constants of the material. Cr-Kα radiation (λ = 2.29 Å) was used. The {211} diffraction plane for the ferrite/martensite phases was used for the analysis. Stress calculations were performed using the standard sin^2^ψ method, assuming a biaxial stress state.

## 3. Results

### 3.1. Microstructural Characterization of the Substrate

**Figure 3:**
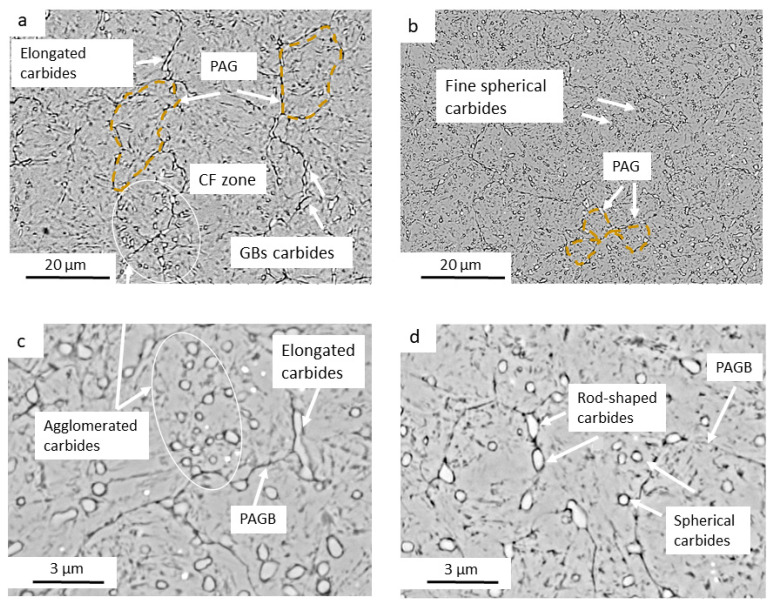
SEM micrographs of the substrate in the tempered state, showing the PAGs and secondary carbides for: (**a**,**c**) SQDT; (**b**,**d**) DQDT.

The microstructural analysis revealed significant differences between the SQDT and DQDT HTs, despite both resulting in a tempered martensitic matrix with M_23_C_6_ carbide precipitation. These differences, illustrated in the SEM micrographs shown in Figure 3, encompass several key aspects of the microstructure, such as the prior austenite grains (PAGs) and the carbides that are known to influence the mechanical properties and performance of the materials.

Firstly, the PAGS showed a marked reduction in the DQDT samples (Figure 3b) compared to the SQDT samples (Figure 3a). The average PAGS in the DQDT samples was measured at 5.7 ± 1.6 µm, compared to 11 ± 3.2 µm in the SQDT samples, representing a nearly 50% reduction. This refinement in grain size is particularly significant, as it aligns with the Hall–Petch relationship [35], which predicts enhanced yield strength and hardness with decreasing grain size. The smaller PAGS in DQDT samples suggests a potential for improved mechanical properties through more effective grain boundary strengthening mechanisms.

Carbide characteristics also differed substantially between the two treatments. In SQDT samples (Figure 3c), carbides were predominantly larger, with an average size of 1.1 ± 0.4 µm, and exhibited an elongated morphology. These carbides showed a strong tendency to nucleate at grain boundaries (GBs), leading to an inhomogeneous distribution characterized by areas of agglomerated carbides interspersed with carbide-free (CF) zones. This heterogeneous distribution can potentially create localized regions of weakness or stress concentration, which may adversely affect the overall mechanical performance. In contrast, the DQDT treatment resulted in a more refined and uniform carbide structure. The average carbide size was reduced to 0.5 ± 0.2 µm, representing a 50% reduction compared to SQDT samples. Moreover, these carbides, shown in Figure 3d, displayed a more spherical morphology and were homogeneously distributed throughout the microstructure. This uniform distribution of finer, more spherical carbides is likely to provide more effective dispersion strengthening while minimizing the stress concentration effects commonly associated with larger, elongated carbides.

**Figure 4:**
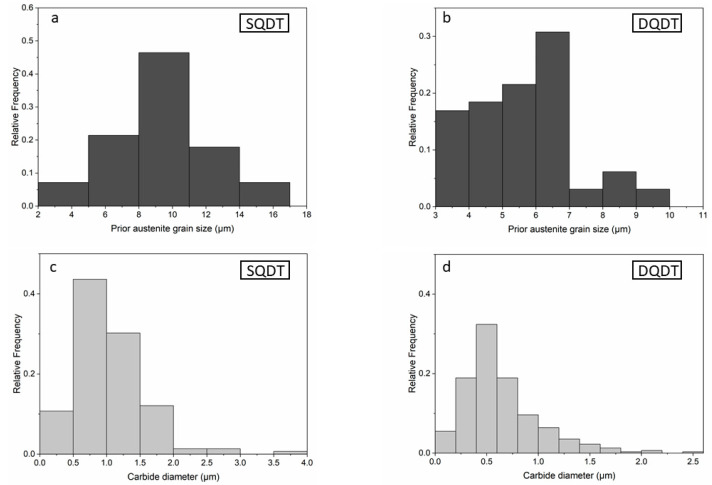
Histogram of the PAGS and carbide size distributions (**a**,**c**) SQDT; (**b**,**d**) DQDT.

Figure 4 presents histogram distributions of PAGS and carbide sizes for both conditions, clearly illustrating the shift towards finer microstructural features in the DQDT sample. The more uniform distribution of carbide sizes in DQDT samples further underscores the enhanced consistency achieved through this treatment. The combination of these microstructural refinements—i.e., smaller PAGS, finer carbides, more uniform carbide distribution, and improved carbide morphology—suggests several potential advantages for knives treated with DQDT.

### 3.2. Field Test Analysis

Following the microstructural analysis, a comprehensive field test was conducted to evaluate the performance of knives treated with SQDT and DQDT HTs under actual industrial operating conditions. The test involved installing the knives in both canter and chipper heads, subjecting them to typical wood processing operations.

**Figure 5:**
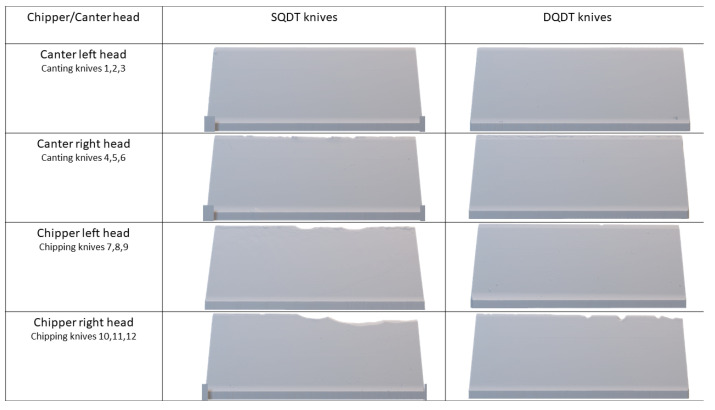
Digital Keyence scans of the most damaged knives in each chipper-canter head for both conditions (SQDT and DQDT).

Figure 5 presents detailed 2D sideview digital microscopic scans of the most damaged knives from each set, displaying eight knives (four for each condition) that represent the most severe damage observed in each canter and chipper head. This visual comparison provides a clear illustration of the contrasting performance between SQDT and DQDT treatments. In the left canter head, both SQDT and DQDT samples showed no visible fractures, indicating similar performance under less demanding conditions. However, in the right canter head, which typically subjects knives to gradual wear, an unexpected and significant fracture was observed in an SQDT sample, challenging conventional expectations. In contrast, DQDT samples in the same right canter head environment showed remarkable resilience, exhibiting no chipping or fracturing. The differences became even more pronounced in the chipper heads, known for their more aggressive and demanding cutting action. In the left chipper head, SQDT samples showed some damage, though less severe than in the right head, while the DQDT sample remarkably displayed no fractures at all. The contrast was most noticeable in the right chipper head, where SQDT knives exhibited severe damage, with a substantial portion of the cutting edge chipped away, indicating a critical failure under high-stress conditions. Conversely, DQDT knives operating in an identical right chipper head environment demonstrated significantly enhanced durability, with only minor chipping observed. This clear contrast in performance across different operational environments strongly suggests that the DQDT treatment confers a significant advantage in terms of wear resistance and structural integrity, particularly under high-stress conditions.

#### 3.2.1. Wear and Volume Loss and in Canting Knives

**Figure 6:**
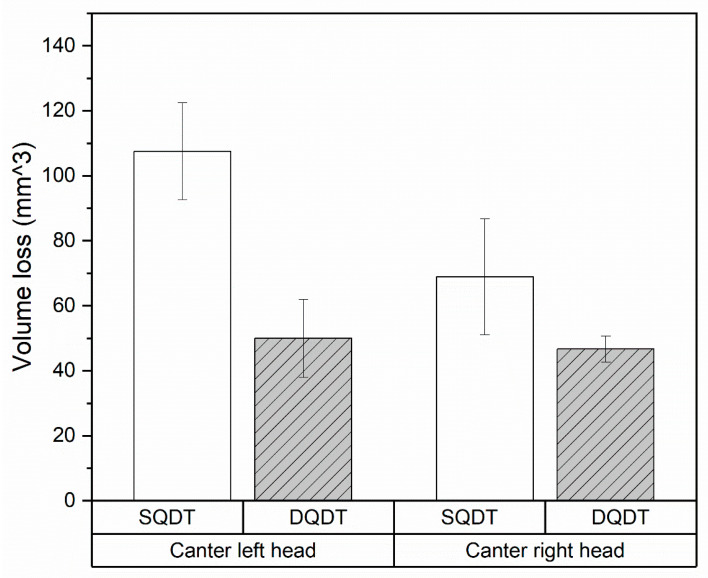
Volume loss in mm^3^ after 124 h of wood cutting in both canter heads.

The analysis of wear behavior focused on the knives installed in the canter heads, which typically experience gradual material loss during wood processing operations. Volume loss measurements were conducted on all knives, with results averaged for each treatment group (SQDT and DQDT) in both the left and right canter heads. This approach allowed for a comprehensive comparison of wear resistance between the two HTs for different operational positions. Figure 6 presents these results in a grouped bar plot, visually illustrating the volume loss for both conditions. In the left canter head, a distinct difference in wear resistance was observed. SQDT knives exhibited an average wear (110 mm^3^) that was nearly 130% higher than that of the DQDT knives (49 mm^3^), indicating that the SQDT knives experienced more than double the material removal under identical operating conditions. The right canter head also demonstrated a clear performance gap, with SQDT samples showing a 50% higher volume loss (70 mm^3^) compared to their DQDT counterparts (46 mm^3^). While this difference is less pronounced than in the left head, it still highlights a substantial improvement in wear resistance for the DQDT knives. Notably, the DQDT samples demonstrated remarkable consistency across both canter heads, with an approximate volume loss of less than 50 mm^3^ in each case. This uniformity suggests that the DQDT treatment provides stable and predictable wear resistance, even when subjected to varying conditions. In contrast, the SQDT samples displayed more variable wear behavior between the two canter heads, with a disparity of almost 130% higher volume loss in the left head versus 50% in the right. This variability indicates that SQDT knives may be more susceptible to fluctuations in operational conditions.

**Figure 7:**
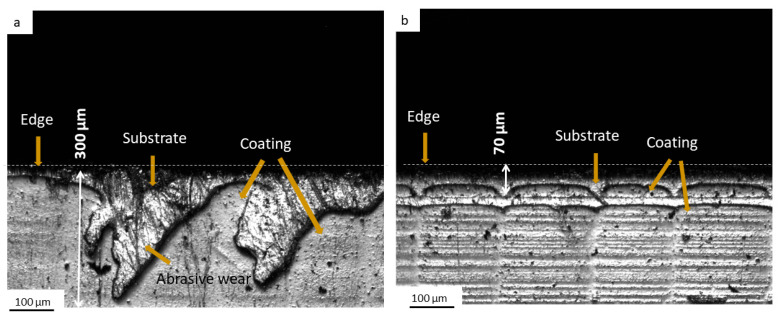
Top-view deferential interference contrast LEXT micrographs of the worn rake surface of the canting knives showing the coating removal: (**a**) SQDT, (**b**) DQDT.

To gain deeper insight into the wear mechanisms at play, laser confocal microscopy was employed to examine the worn edges of both SQDT- and DQDT-treated knives. Figure 7 presents micrographs of representative samples from each condition, revealing visible differences in their wear patterns. In the SQDT sample, extensive removal of the PVD coating is evident, extending up to 300 μm below the knife edge, as shown in Figure 7a. This substantial loss of protective coating has left the underlying substrate exposed to direct contact with the wood material, resulting in pronounced abrasive wear along the SQDT knife edge. The wear pattern suggests that once the PVD coating was compromised, the softer substrate experienced accelerated material removal. In contrast, the DQDT sample exhibits remarkably better coating retention. The PVD coating on the DQDT knife remains largely intact, with a depth of abrasion of only 70 μm, showing signs of coating detachment near the very edge, as illustrated in Figure 7b. This superior coating adhesion in the DQDT sample has effectively protected the substrate from extensive abrasive wear, as evidenced by the absence of the pronounced wear marks observed in the SQDT sample. The notable difference in coating retention and subsequent wear behavior between the two samples visually supports the volume loss measurements and provides valuable insights into the mechanisms underlying the enhanced wear resistance of the DQDT knives.

#### 3.2.2. Damage and Edge Stability in the Chipping Knives

**Figure 8:**
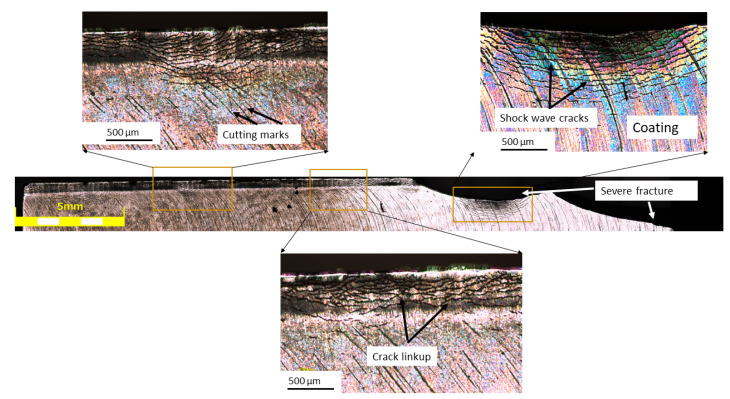
Stitched LEXT micrography of the SQDT knife edge with corresponding enlargement zones.

The failure analysis of the edges focused on the most severely affected knives (shown in Figure 5) from the chipper head, with particular attention given to the SQDT sample that exhibited the most extensive fracturing. Figure 8 presents a comprehensive view of the damage, featuring a stitched micrograph composed of 15 images of the reconstructed edge obtained using the LEXT microscope, alongside enlarged views of specific zones of interest. The stitched micrograph provides a panoramic view of the edge, revealing a complex pattern of damage along its length. It must be noted that, despite the severe damage, portions of the PVD coating remained adherent to the knife edge, indicating that coating detachment was not uniform across the blade. Moreover, development of cracks along the edge, with varying degrees of severity, and patterns are visible on the surface of the samples. An important observation is that these cracks frequently intersected the cutting marks on the knife edge (Figure 8, top left inset). This intersection suggests a strong interaction between the operational stresses and the existing surface features, potentially indicating that the cutting marks could act as stress concentrators or crack initiation sites. In certain regions, as shown in the enlarged inset (Figure 8, bottom right inset), the crack propagation was particularly pronounced, leading to observable crack link-up. This phenomenon suggests that under high stress, multiple cracks are initiated, often at these cutting mark intersections, and eventually coalesce, leading to material removal. An intriguing aspect of the crack behavior, visible in the top right inset, was their propagation pattern, which resembled shockwaves. This distinctive pattern was more prominently visible in the coating layer, possibly due to its more brittle nature compared to the substrate, thus providing a clearer visualization of the crack propagation paths. The interaction between these shockwave-like cracks and the cutting marks further emphasizes the complex stress state at the knife edge during operation.

In some areas, the cracks appear to propagate both parallel and perpendicular to the edge, creating a network of intersecting fracture lines. This complex crack network, often initiating or redirecting at cutting mark intersections, suggests that the knife edge was subjected to multidirectional stresses during operation, leading to a complicated failure mechanism. The persistence of the coating in some areas, coupled with the complex crack patterns and their interaction with cutting marks, suggests a multifaceted damage mechanism involving the coating, the substrate material, and the surface topography created during operation. These observations provide valuable insights into the failure mode of SQDT knives under the high-stress conditions of the chipper head. The varying crack morphologies, their propagation patterns, and their relationship with cutting marks indicate that the damage evolution is not uniform along the edge, likely influenced by the combination of mechanical and microstructural features.

**Figure 9:**
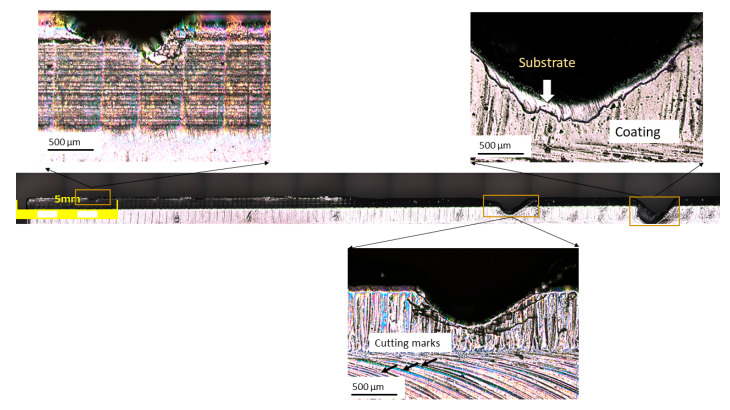
Stitched LEXT micrography of the DQDT knife edge with corresponding enlargement zones.

On the other hand, the analysis of the most damaged knife from the DQDT sample revealed a significantly better condition compared to its SQDT counterpart. Figure 9 presents a stitched micrograph of the knife edge, along with enlarged insets highlighting small chipped regions. Notably, the PVD coating remained largely intact on this knife, highlighting the effectiveness of the DQDT treatment in preserving the coating under operational conditions. The micrograph shows only a single crack present in a localized area (Figure 9, bottom right inset), which appears to be the result of a decohesion between the PVD coating and the substrate. Importantly, the small chipped regions, as illustrated in the insets (top right and top left) in Figure 9, do not compromise the cutting ability of the knife, even after 124 h of operation. The limited extent of damage indicates that the knife retains its functional performance, allowing it to maintain efficient cutting action despite the challenging operational environment. This minimal damage contrasts sharply with the extensive fractures observed in the SQDT sample, indicating that the DQDT knife experienced far less wear and damage during its time in the chipper head. The presence of the coating in most areas suggests that the DQDT treatment not only enhances the knife’s wear resistance but also contributes to the integrity of the protective coating, effectively shielding the underlying material from the abrasive forces encountered during operation.

#### 3.2.3. Fracture Surface of Chipping Knives

**Figure 10:**
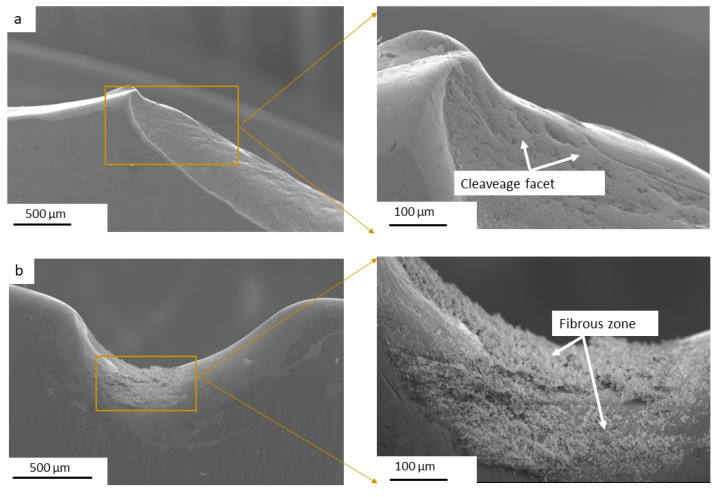
SEM images of the fractured chipped areas for both conditions: (**a**) SQDT, (**b**) DQDT.

The fractography analysis was performed using SEM with secondary electron imaging to examine the fracture surfaces of the most damaged knives from both the SQDT and DQDT treatments. Figure 10 presents the SEM images highlighting the distinct fracture characteristics observed in each knife. In the SQDT knife presented in Figure 10a, the fracture surface exhibited a brittle failure mode, characterized by a quasi-cleavage appearance. This type of fracture is indicative of rapid crack propagation with minimal plastic deformation, suggesting that the material failed under high-stress conditions without significant energy absorption. In contrast, the DQDT knife displayed a markedly different fracture profile in the chipped area, as shown in Figure 10b, revealing a fibrous appearance characterized by the presence of dimples. These dimples are indicative of ductile fracture behavior, which suggests that plastic deformation occurred prior to failure. The presence of such dimples indicates that the DQDT knife was able to undergo some degree of deformation, absorbing energy and thereby delaying catastrophic failure.

#### 3.2.4. Crack Initiation and Propagation

**Figure 11:**
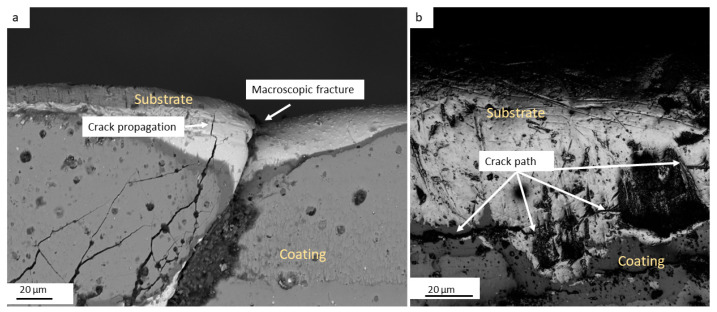
SEM micrography illustrating the crack path near the edge in the SQDT sample: (**a**) a crack propagating from the substrate to the PVD coating; (**b**) PVD decohesion due to crack propagation.

The analysis of crack initiation and propagation was conducted on the SQDT knife, which exhibited a notably higher density of cracks compared to the DQDT sample. This examination revealed a distinct mechanism whereby cracks predominantly initiated from the substrate and propagated through the PVD coating, indicating a critical interaction between the substrate material and the protective layer. In Figure 11, two micrographs effectively illustrate this phenomenon: Figure 11a captures a significant macroscopic fracture that extends from the edge of the knife down into the PVD coating. Adjacent to this fracture, another crack originating in the substrate, which propagates toward the PVD coating, is illustrated. As this crack approaches the interface between the steel substrate and the PVD coating, it becomes evident that the fracture behavior changes. Upon reaching the interface, secondary cracks emerge within the coating itself, albeit with lower intensity than the primary crack propagating from the substrate. This observation suggests that the interface between the substrate and the coating is a critical zone for crack propagation, where the mechanical properties of both materials influence the overall fracture behavior. The formation of these secondary cracks indicates that the PVD coating, while providing some degree of protection, is not fully capable of withstanding the stresses transmitted from the substrate once primary cracks initiate. Figure 11b further elucidates the consequences of this crack propagation. It can be seen that the advancing crack in the substrate leads to decohesion of the PVD coating. This decohesion is particularly concerning, as it not only compromises the integrity of the protective layer but also facilitates the propagation of cracks into the coating, potentially leading to more extensive damage. The microscopic examination of these samples revealed that as the primary crack advances, it disrupts the bond at the interface, resulting in localized failure of the coating. This failure can create pathways for further crack growth, exacerbating the material’s vulnerability to wear and reducing its overall performance.

**Figure 12:**
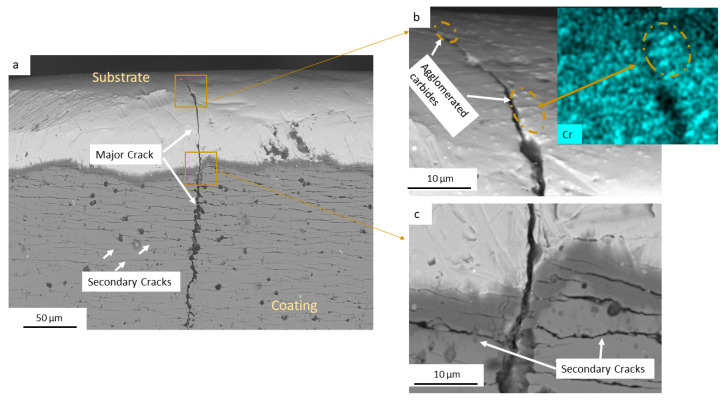
(**a**) SEM micrograph showing crack propagation from the substrate toward the PVD coating; (**b**) enlargement of the crack path along with EDS analysis of Cr; (**c**) enlargement of the interface substrate/coating and secondary crack nucleation.

Figure 12a presents a micrograph of the crack, accompanied by an enlargement of the initiation zone and an energy-dispersive spectroscopy (EDS) analysis of Cr content in Figure 12b. It can be seen that the crack begins near agglomerated M_23_C_6_ carbide particles, indicating that these carbides play a significant role in the crack initiation process. Notably, the crack path appears to progress from one carbide to another, suggesting that the presence of these agglomerated particles creates localized stress concentrations that facilitate crack growth. The EDS analysis, shown in Figure 12b, further supports this observation, as it highlights the composition of the agglomerated particles, which are mainly composed of Cr_23_C_6_ carbides. This relationship between the crack initiation and the agglomerated Cr-rich carbides reveals the importance of controlling the size and distribution of carbides in this steel.

Figure 12c shows the interface between the substrate and the PVD coating, illustrating how secondary cracks form from the main crack. These secondary cracks develop as branches within the coating, reflecting the complex stress state experienced during operation. Given that the crack propagates through M_23_C_6_ carbides in the substrate, and considering that these carbides predominantly nucleate at grain boundaries (see Section 3.1), it is plausible to suggest that the crack primarily propagates along these boundaries, resulting in an intergranular fracture mode, as observed in SQDT knives.

### 3.3. Residual Stress Analysis

**Figure 13:**
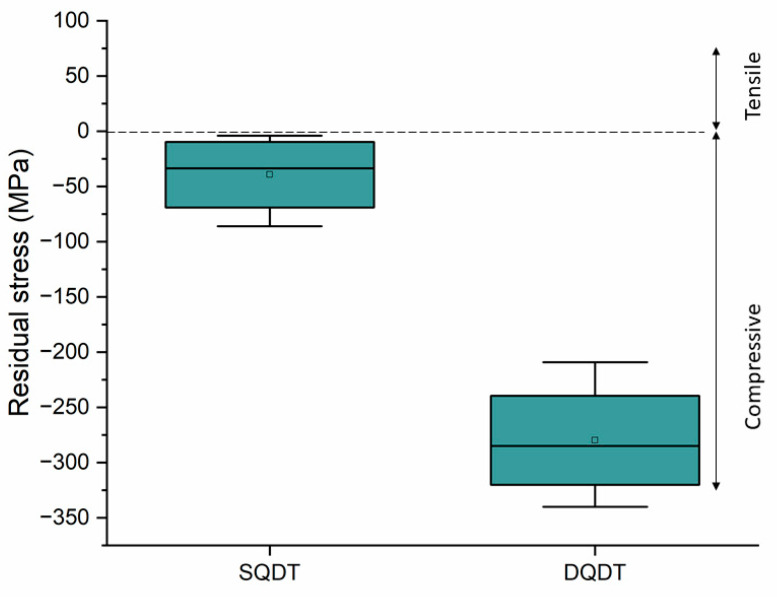
Box plot of the residual stress measurement after HT and prior to the field test for SQDT and DQDT samples.

Given the marked difference in wear and fracture behavior between the two treatments, residual stress measurements were conducted on both SQDT and DQDT knives before and after the field test, and the results are reported in Figure 13.

The results show that, prior to the field tests, the SQDT samples exhibited an average compressive residual stress of approximately −30 MPa, while the DQDT samples displayed a significantly higher average compressive residual stress of around −280 MPa. The enhanced compressive residual stresses in the DQDT samples are likely to contribute to their improved resistance to crack initiation and propagation [36,37]. In contrast, the lower compressive residual stresses in the SQDT samples may render them more susceptible to crack propagation, as the internal stresses could facilitate the development of fractures under operational conditions.

**Figure 14:**
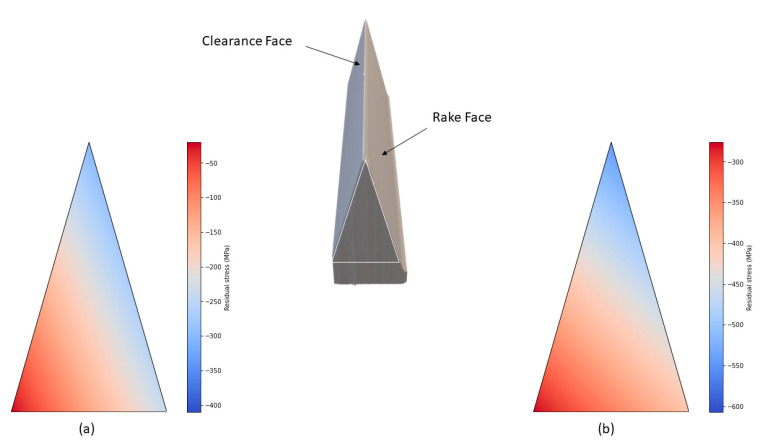
Thermal map showing the dispersion of the residual stresses near the edge after wood machining: (**a**) SQDT knife and (**b**) DQDT knife. The triangle with a white line boundary represents the projection of the measured surface.

The analysis of residual stresses after the field test provides critical insights into the performance of the SQDT and DQDT knives. Following the tests, residual stresses were measured at various depths from the edge, specifically on the rake surface (where the knife is in contact with the wood) and on the clearance surface. A thermal map was generated to illustrate how these residual stresses evolved during the wood machining process and is presented in Figure 14.

Wood cutting in chipper–canter heads has been considered as an orthogonal cutting process where the chips apply a thermomechanical (pressure and heat) load on the rake face [38]. The results reported in Figure 14 indicate that for the SQDT samples, the compressive residual stresses near the edge increased to approximately −300 MPa, while in regions not in contact with the wood, the stresses were around −40 MPa, near the values measured before the test. In contrast, the DQDT samples exhibited a more significant increase in compressive residual stresses, reaching nearly −550 MPa at the edge, and decreasing to about −300 MPa in the zones not in contact with the wood. These findings suggest that although the field test increased the compressive residual stresses on the surfaces of both treatments, the enhancement was not sufficient to mitigate crack propagation in the SQDT samples.

## 4. Discussion

The literature has consistently suggested that the presence of secondary carbides in tool steels has a notable impact on the mechanical properties. The interfaces between secondary hard particles and the steel substrate are considered as high stress concentrator zones [39], and cracks could easily initiate at these locations. Furthermore, the presence of agglomerated carbides in specific regions could dictate the crack path behavior [40]. Larrin [3] suggested that reducing the carbide size near the edge is an effective way to diminish edge chipping. In this context, the microstructural refinement achieved through the DQDT cycle is particularly noteworthy, as it is expected to increase the grain boundary density and especially the high-angle boundaries [26,41]. The latter play a vital role in impeding crack propagation and enhancing toughness [42,43]. A higher density of high-angle boundaries could also provide more nucleation sites for carbides [44,45]. The obtained results show that, in the case of DQDT, the preferential nucleation sites for carbides are effectively optimized, resulting in a predominance of spherical carbides that are homogeneously distributed throughout the matrix. In contrast, the SQDT knives exhibit larger, more elongated carbides, which often result in an inhomogeneous distribution, increasing their susceptibility to wear and chipping. Furthermore, the spherical morphology of the carbides in DQDT contributed to improved dispersion strengthening [46] and reduced stress concentration points, thereby enhancing the overall durability of the cutting edge.

The relationship between microstructure refinement and the adherence of PVD coatings is critical in enhancing the performance of cutting tools. Research indicates that smaller grain sizes in the substrate enhance the mechanical properties of the PVD coating [47], as they could provide more nucleation sites for coating layers, which can significantly improve the adhesion strength of the coating. The presence of hard secondary particles in the substrate could also improve the adhesion between the layers and the substrate [48]. DQDT treatment resulted in microstructural refinement, increasing the density of grain boundaries, particularly high-angle grain boundaries. This refinement not only enhances the mechanical properties of the substrate but also creates an optimal environment for the nucleation of PVD coating layers. Consequently, DQDT samples exhibit more uniform and robust adhesion of the PVD coating compared to SQDT knives. The refined microstructure, with its numerous nucleation sites, facilitates better interlocking and mechanical anchoring of the coating, thereby improving its durability and wear resistance. In contrast, SQDT knives, with their larger grains and less favorable grain boundary distribution, tend to have weaker bonding at the coating–substrate interface.

Finally, a finer microstructure leads often to higher compressive residual stress due to the increased density of grain boundaries and dislocations [49], which act as barriers to the movement of defects and enable more effective strain accommodation. When the microstructure is refined, the smaller grain sizes and higher dislocation density introduce greater strain energy in the material. In the context of knife cutting tools and blades, introducing compressive residual stresses near the edge through mechanical peening has been reported to enhance the tool lifespan and prevent the edge from chipping [50,51].

The accumulation of compressive residual stresses in the surface has shown great benefits for PVD coating adhesion as well [52]. High compressive residual stress helps to counterbalance the tensile stress that develops in the coating during the deposition process, reducing the likelihood of coating delamination or cracking [53].

## 5. Conclusions

In the present work the performance of modified AISI A8 tool steel knives used in wood cutting applications was evaluated by applying single quenching and double tempering (SQDT) and double quenching and double tempering (DQDT) treatments. The key findings are as follows:

- DQDT treatment resulted in a finer microstructure, with a reduction in the PAGS and carbide size up to 50%, and a more uniform and spherical carbide distribution compared to SQDT treatment.
- Field tests revealed that DQDT knives exhibited superior wear resistance, with improvements of up to 130% over SQDT knives.
- Fractography analysis revealed a transition from brittle fractures with quasi-cleavage and severe crack linkups in SQDT knives to ductile fractures characterized by dimples in DQDT knives.
- Residual stress measurements indicated that the newly proposed DQDT treatment introduced higher compressive stresses (−280 MPa) compared to SQDT (−30 MPa), which correlated with improved performance and better preservation of the PVD coating.

Overall, these findings underscore the effectiveness of DQDT in significantly enhancing the durability and efficiency of wood cutting tools, providing valuable insights for optimizing heat treatment processes in the wood processing industry.

## Figures and Tables

**Table 1:** Chemical composition of the modified A8 tool steel.

Element	C	Si	Cr	Mn	Mo	Ni	Fe
Wt. %	0.40	0.80	8.00	0.40	1.20	0.14	Bal.

## Data Availability

The original contributions presented in the study are included in the article, further inquiries can be directed to the corresponding authors.
